# Supplementary material for: Pyruvate Dehydrogenase Kinase 1 inhibition mediated oxidative phosphorylation enhancement in cartilage promotes osteoarthritis progression
Source: BMC Musculoskelet Disord. 2023 Jul 20;24:597. doi: 10.1186/s12891-023-06585-6 (PMC10357736; doi:10.1186/s12891-023-06585-6)
Supplement: Supplementary file 1 — Additional file 1. [file 12891_2023_6585_MOESM1_ESM.zip › BP.Gsea.1653623667859/GOBP_NUCLEOSIDE_METABOLIC_PROCESS.html]

Details for gene set GOBP\_NUCLEOSIDE\_METABOLIC\_PROCESS[GSEA]

|  || Dataset | 55457\_collapsed\_to\_symbols.cls.cls #OA\_versus\_normal.cls.cls #OA\_versus\_normal\_repos |
| Phenotype | cls.cls#OA\_versus\_normal\_repos |
| Upregulated in class | OA |
| GeneSet | GOBP\_NUCLEOSIDE\_METABOLIC\_PROCESS |
| Enrichment Score (ES) | 0.38157114 |
| Normalized Enrichment Score (NES) | 1.5627347 |
| Nominal p-value | 0.007614213 |
| FDR q-value | 0.2826841 |
| FWER p-Value | 1.0 |
Table: GSEA Results Summary

  

Fig 1: Enrichment plot: GOBP\_NUCLEOSIDE\_METABOLIC\_PROCESS      
 Profile of the Running ES Score & Positions of GeneSet Members on the Rank Ordered List

  

| SYMBOL | TITLE | RANK IN GENE LIST | RANK METRIC SCORE | RUNNING ES | CORE ENRICHMENT || 1 | DGUOK | deoxyguanosine kinase [Source:HGNC Symbol;Acc:HGNC:2858] | 96 | 0.917 | 0.0463 | Yes |
| 2 | ADK | adenosine kinase [Source:HGNC Symbol;Acc:HGNC:257] | 127 | 0.878 | 0.0956 | Yes |
| 3 | NME5 | NME/NM23 family member 5 [Source:HGNC Symbol;Acc:HGNC:7853] | 362 | 0.720 | 0.1196 | Yes |
| 4 | ATIC | 5-aminoimidazole-4-carboxamide ribonucleotide formyltransferase/IMP cyclohydrolase [Source:HGNC Symbol;Acc:HGNC:794] | 401 | 0.706 | 0.1581 | Yes |
| 5 | NME4 | NME/NM23 nucleoside diphosphate kinase 4 [Source:HGNC Symbol;Acc:HGNC:7852] | 677 | 0.599 | 0.1718 | Yes |
| 6 | DNPH1 | 2'-deoxynucleoside 5'-phosphate N-hydrolase 1 [Source:HGNC Symbol;Acc:HGNC:21218] | 719 | 0.589 | 0.2032 | Yes |
| 7 | ENPP4 | ectonucleotide pyrophosphatase/phosphodiesterase 4 [Source:HGNC Symbol;Acc:HGNC:3359] | 822 | 0.563 | 0.2283 | Yes |
| 8 | DERA | deoxyribose-phosphate aldolase [Source:HGNC Symbol;Acc:HGNC:24269] | 975 | 0.523 | 0.2472 | Yes |
| 9 | ICMT | isoprenylcysteine carboxyl methyltransferase [Source:HGNC Symbol;Acc:HGNC:5350] | 1140 | 0.488 | 0.2630 | Yes |
| 10 | APRT | adenine phosphoribosyltransferase [Source:HGNC Symbol;Acc:HGNC:626] | 1178 | 0.481 | 0.2884 | Yes |
| 11 | APOBEC3C | apolipoprotein B mRNA editing enzyme catalytic subunit 3C [Source:HGNC Symbol;Acc:HGNC:17353] | 1355 | 0.450 | 0.3011 | Yes |
| 12 | NUDT1 | nudix hydrolase 1 [Source:HGNC Symbol;Acc:HGNC:8048] | 1425 | 0.439 | 0.3215 | Yes |
| 13 | AICDA | activation induced cytidine deaminase [Source:HGNC Symbol;Acc:HGNC:13203] | 1825 | 0.381 | 0.3128 | Yes |
| 14 | PTGDR | prostaglandin D2 receptor [Source:HGNC Symbol;Acc:HGNC:9591] | 1904 | 0.372 | 0.3286 | Yes |
| 15 | APOBEC3G | apolipoprotein B mRNA editing enzyme catalytic subunit 3G [Source:HGNC Symbol;Acc:HGNC:17357] | 1917 | 0.370 | 0.3494 | Yes |
| 16 | ADA2 | adenosine deaminase 2 [Source:HGNC Symbol;Acc:HGNC:1839] | 1955 | 0.367 | 0.3680 | Yes |
| 17 | APOBEC1 | apolipoprotein B mRNA editing enzyme catalytic subunit 1 [Source:HGNC Symbol;Acc:HGNC:604] | 2695 | 0.280 | 0.3269 | Yes |
| 18 | OARD1 | O-acyl-ADP-ribose deacylase 1 [Source:HGNC Symbol;Acc:HGNC:21257] | 2713 | 0.278 | 0.3419 | Yes |
| 19 | PRPSAP2 | phosphoribosyl pyrophosphate synthetase associated protein 2 [Source:HGNC Symbol;Acc:HGNC:9467] | 2855 | 0.265 | 0.3465 | Yes |
| 20 | TK1 | thymidine kinase 1 [Source:HGNC Symbol;Acc:HGNC:11830] | 2909 | 0.259 | 0.3576 | Yes |
| 21 | UMPS | uridine monophosphate synthetase [Source:HGNC Symbol;Acc:HGNC:12563] | 2936 | 0.257 | 0.3706 | Yes |
| 22 | MTAP | methylthioadenosine phosphorylase [Source:HGNC Symbol;Acc:HGNC:7413] | 3324 | 0.219 | 0.3533 | Yes |
| 23 | NT5E | 5'-nucleotidase ecto [Source:HGNC Symbol;Acc:HGNC:8021] | 3515 | 0.201 | 0.3503 | Yes |
| 24 | DTYMK | deoxythymidylate kinase [Source:HGNC Symbol;Acc:HGNC:3061] | 3538 | 0.199 | 0.3603 | Yes |
| 25 | TYMP | thymidine phosphorylase [Source:HGNC Symbol;Acc:HGNC:3148] | 3588 | 0.195 | 0.3679 | Yes |
| 26 | APOBEC3F | apolipoprotein B mRNA editing enzyme catalytic subunit 3F [Source:HGNC Symbol;Acc:HGNC:17356] | 3603 | 0.193 | 0.3781 | Yes |
| 27 | PRPS1 | phosphoribosyl pyrophosphate synthetase 1 [Source:HGNC Symbol;Acc:HGNC:9462] | 3717 | 0.184 | 0.3801 | Yes |
| 28 | DPYD | dihydropyrimidine dehydrogenase [Source:HGNC Symbol;Acc:HGNC:3012] | 3830 | 0.173 | 0.3816 | Yes |
| 29 | APOBEC2 | apolipoprotein B mRNA editing enzyme catalytic subunit 2 [Source:HGNC Symbol;Acc:HGNC:605] | 4279 | 0.130 | 0.3543 | No |
| 30 | PRPS2 | phosphoribosyl pyrophosphate synthetase 2 [Source:HGNC Symbol;Acc:HGNC:9465] | 4365 | 0.123 | 0.3549 | No |
| 31 | MACROD1 | mono-ADP ribosylhydrolase 1 [Source:HGNC Symbol;Acc:HGNC:29598] | 5008 | 0.072 | 0.3092 | No |
| 32 | APOBEC3B | apolipoprotein B mRNA editing enzyme catalytic subunit 3B [Source:HGNC Symbol;Acc:HGNC:17352] | 5089 | 0.065 | 0.3068 | No |
| 33 | ERH | ERH mRNA splicing and mitosis factor [Source:HGNC Symbol;Acc:HGNC:3447] | 5109 | 0.063 | 0.3091 | No |
| 34 | ACP3 | acid phosphatase 3 [Source:HGNC Symbol;Acc:HGNC:125] | 6271 | -0.025 | 0.2202 | No |
| 35 | HPRT1 | hypoxanthine phosphoribosyltransferase 1 [Source:HGNC Symbol;Acc:HGNC:5157] | 6954 | -0.076 | 0.1716 | No |
| 36 | TK2 | thymidine kinase 2 [Source:HGNC Symbol;Acc:HGNC:11831] | 7468 | -0.116 | 0.1385 | No |
| 37 | ADA | adenosine deaminase [Source:HGNC Symbol;Acc:HGNC:186] | 8105 | -0.163 | 0.0986 | No |
| 38 | CDA | cytidine deaminase [Source:HGNC Symbol;Acc:HGNC:1712] | 8324 | -0.180 | 0.0922 | No |
| 39 | XDH | xanthine dehydrogenase [Source:HGNC Symbol;Acc:HGNC:12805] | 8366 | -0.183 | 0.0997 | No |
| 40 | PRPS1L1 | phosphoribosyl pyrophosphate synthetase 1 like 1 [Source:HGNC Symbol;Acc:HGNC:9463] | 8382 | -0.184 | 0.1094 | No |
| 41 | GNMT | glycine N-methyltransferase [Source:HGNC Symbol;Acc:HGNC:4415] | 9208 | -0.248 | 0.0597 | No |
| 42 | NT5C2 | "5'-nucleotidase, cytosolic II [Source:HGNC Symbol;Acc:HGNC:8022]" | 9595 | -0.283 | 0.0463 | No |
| 43 | UPB1 | beta-ureidopropionase 1 [Source:HGNC Symbol;Acc:HGNC:16297] | 9609 | -0.284 | 0.0620 | No |
| 44 | PNP | purine nucleoside phosphorylase [Source:HGNC Symbol;Acc:HGNC:7892] | 9657 | -0.288 | 0.0752 | No |
| 45 | AMD1 | adenosylmethionine decarboxylase 1 [Source:HGNC Symbol;Acc:HGNC:457] | 10415 | -0.363 | 0.0376 | No |
| 46 | APOBEC3A | apolipoprotein B mRNA editing enzyme catalytic subunit 3A [Source:HGNC Symbol;Acc:HGNC:17343] | 10550 | -0.378 | 0.0493 | No |
| 47 | UCKL1 | uridine-cytidine kinase 1 like 1 [Source:HGNC Symbol;Acc:HGNC:15938] | 11515 | -0.503 | 0.0038 | No |
| 48 | PPAT | phosphoribosyl pyrophosphate amidotransferase [Source:HGNC Symbol;Acc:HGNC:9238] | 11534 | -0.505 | 0.0320 | No |
| 49 | UPP1 | uridine phosphorylase 1 [Source:HGNC Symbol;Acc:HGNC:12576] | 12013 | -0.595 | 0.0298 | No |
| 50 | CDADC1 | cytidine and dCMP deaminase domain containing 1 [Source:HGNC Symbol;Acc:HGNC:20299] | 12267 | -0.667 | 0.0493 | No |
Table: GSEA details [plain text format]

  

Fig 2: GOBP\_NUCLEOSIDE\_METABOLIC\_PROCESS      
 Blue-Pink O' Gram in the Space of the Analyzed GeneSet

  

Fig 3: GOBP\_NUCLEOSIDE\_METABOLIC\_PROCESS: Random ES distribution      
 Gene set null distribution of ES for **GOBP\_NUCLEOSIDE\_METABOLIC\_PROCESS**
